# Characterization and genomic analysis of a diesel-degrading bacterium, *Acinetobacter calcoaceticus* CA16, isolated from Canadian soil

**DOI:** 10.1186/s12896-020-00632-z

**Published:** 2020-07-25

**Authors:** Margaret T. Ho, Michelle S. M. Li, Tim McDowell, Jacqueline MacDonald, Ze-Chun Yuan

**Affiliations:** 1 Department of Microbiology and Immunology, The University of Western Ontario, 1151 Richmond Street, London, Ontario N6A 5B7 Canada; 2 Institute of Biomaterials and Biomedical Engineering, University of Toronto, Toronto, Ontario Canada; 3 London Research and Development Centre, Agriculture and Agri-Food Canada, 1391 Sandford Street, London, Ontario N5V 4T3 Canada

**Keywords:** Microbial bioremediation, *Acinetobacter calcoaceticus* CA16, Diesel-degrading bacteria, Diesel bioremediation, Aliphatic hydrocarbons, *n*-alkanes

## Abstract

**Background:**

With the high demand for diesel across the world, environmental decontamination from its improper usage, storage and accidental spills becomes necessary. One highly environmentally friendly and cost-effective decontamination method is to utilize diesel-degrading microbes as a means for bioremediation. Here, we present a newly isolated and identified strain of *Acinetobacter calcoaceticus* (‘CA16’) as a candidate for the bioremediation of diesel-contaminated areas.

**Results:**

*Acinetobacter calcoaceticus* CA16 was able to survive and grow in minimal medium with diesel as the only source of carbon. We determined through metabolomics that *A. calcoaceticus* CA16 appears to be efficient at diesel degradation. Specifically, CA16 is able to degrade 82 to 92% of aliphatic alkane hydrocarbons (C_*n*_H_*n* + 2_; where *n* = 12–18) in 28 days. Several diesel-degrading genes (such as *alk*M and *xcp*R) that are present in other microbes were also found to be activated in CA16.

**Conclusions:**

The results presented here suggest that *Acinetobacter* strain CA16 has good potential in the bioremediation of diesel-polluted environments.

## Background

With the high demand for diesel around the world, severe environmental and ecological problems have arisen from its improper usage, storage and disposal, as well as accidental leakage. Diesel oil, a very complex mixture of hydrocarbons (e.g. aliphatics, aromatics, alcohols), and its byproducts, are known soil contaminants and are phytotoxic to a wide variety of plants [1, 2]. Such effects can be mitigated by microbial bioremediation, which uses microbes to remove pollutants from the environment [3, 4]. In particular, bioaugmentation involves the addition of living bacteria to a contaminated site, while biostimulation adds supplemental nutrients to existing on-site bacteria with the goal of optimizing their metabolism [5]. On-site bioremediation is considered one of the cheapest and least laborious methods to remove unwanted hydrocarbons from contaminated ecosystems, and it is also effective and environmentally friendly [6]. Sites can be pre-screened for diesel-degrading bacterial candidates that can promote plant growth [7] and reduce diesel-induced phytotoxicity [8] in these contaminated areas.

Ultimately, diesel degradation by microorganisms varies and depends highly on the microbe’s ability to utilize the hydrocarbon components or (by) products of diesel degradation [8, 9]. Some microbes, such as *Alcanivorax borkumensis,* which is often considered the main diesel degrader, can degrade various *n-*alkanes that are present in diesel [6, 10–12]. On the other hand, many microbes can only degrade a single hydrocarbon class (e.g. aromatics vs. aliphatics) or hydrocarbon component of diesel. Because the ability to degrade multiple components of the contaminant is often limited with single strains, many research groups are using multiple microbial stains together as a consortium [13]. The consortia are chosen based on the individual strain’s properties and ability to work synergistically with other microbes to promote higher overall degradation efficiency. It is therefore important to study a variety of microbes, and even those with overlapping or repetitive abilities in terms of degradation may hold different values in different situations depending on soil characteristics and other prevailing environmental factors.

Members of the *Acinetobacter* genus have previously been shown to degrade diesel components [14, 15]. In fact, some *Acinetobacter* strains have the ability to degrade a broad variety of hydrocarbons; such as the *n-*alkanes [16–18] and aromatics [19, 20]. In addition, *Acinetobacter sp.* thrive very well under extreme environmental conditions, such as high altitude lakes, dry surfaces, alkaline or hyper-saline environments, both cold and high temperatures, oil-contaminated soil, as well as in organic solvents [21–25]. We suspect the *Acinetobacter* genus may offer a solution or play a role in bioremediation in a variety of environments, including remote and harsh ecosystems.

In North America, increasing exports of some petroleum products from Canada to the United States since 2018, via pipelines and rail, has increased the risk of terrestrial oil spills in these countries [26]. In urban areas of Southern Ontario, Canada, fuel tank and barrel leaks have also contributed 11.1% of more than 700 recorded chemical spills per year, most of which have been cleaned up only partially, or not at all [27]. For such terrestrial spills, it is known that the success of bioremediation is dependent on local factors such as soil type and climate [26]. Therefore, research into the petroleum-degrading abilities of locally isolated soil bacteria may lead to the development of strains or consortia for bioremediation of these contaminated sites.

Here, we characterized an *Acinetobacter calcoaceticus* strain (‘CA16’) with a completely sequenced genome, which was isolated in London, Ontario, Canada, and examined its diesel-degrading capabilities under in vitro conditions. Metabolomics was used to study changes in hydrocarbon concentration following treatment with CA16, while quantitative reverse-transcription polymerase chain reaction (qRT-PCR) confirmed expression of genes known to be involved in diesel degradation. Plant growth-promoting characteristics were also assessed in a preliminary manner, and will be further characterized in the future, as these traits could help the establishment or recovery of vegetation at a contaminated site. Overall, insights from this study are expected to establish CA16’s place in a catalog of strains with potential application to petroleum bioremediation of Canadian soils. To our knowledge, strain CA16 is the first diesel-degrading *Acinetobacter* to be isolated from Canada. In addition, it is the first and only strain of *A. calcoaceticus* with both a completed genome sequence and proven diesel-degrading ability.

## Results

### Isolation and screening of bacterial isolate CA16

Bacterial isolates, consisting of white raised and concave colonies, were isolated from canola roots. These isolates were screened in minimal medium supplemented with 0.2% diesel as the only carbon source to assess their diesel-degrading properties. The isolate that had the largest colony and fastest growth rate in diesel-supplemented medium was named CA16. Based on the optical density data, this bacterial isolate grew optimally at 28 °C and a pH of 6.0 (data not shown). In addition to diesel degradation, our preliminary analyses indicate that CA16 is capable of nitrogen fixation, phosphate and potassium solubilization, and utilizing lignin as a sole carbon source. However, the isolate was incapable of fixing carbon dioxide and did not produce nanocellulose (data not shown). Further research to confirm and quantify these capabilities will be presented in a subsequent manuscript.

### Genomic sequencing and phylogenetic analysis

**Fig. 1:**
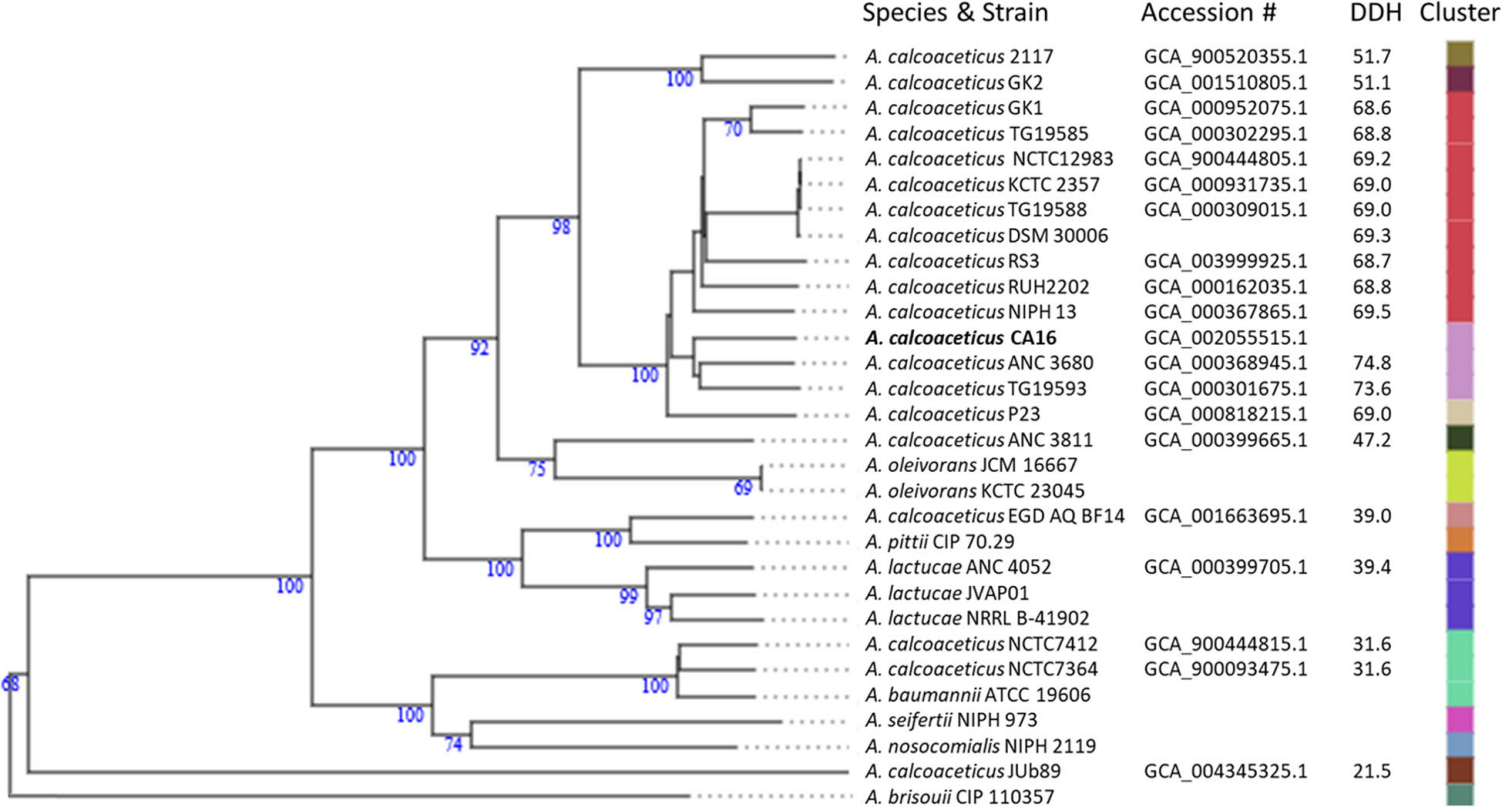
Phylogenetic tree illustrates that CA16 clusters with strains of *A. calcoaceticus.* The accession numbers for non-type strains are from the NCBI. DDH values are between the indicated strain and CA16. Strains with the same color in the “cluster” column are predicted to be of the same species based on a DDH value of ≥70%. The tree was inferred with FastME 2.1.6.1 from the GBDP distances calculated based on their genome sequences. The branch lengths are scaled in terms of GBDP distance formula d5. The numbers above branches are GBDP pseudo-bootstrap support values > 60% from 100 replications, with an average branch support of 68.2%. The tree was trimmed to include only the Acb complex species with *Acinetobacter brisouii* as the outgroup

Whole-genome comparison identified that the bacterial type strain most closely related to CA16 is *A. calcoaceticus* DSM 30006. A phylogenetic tree including CA16, DSM 30006 and 18 other sequenced strains of *A. calcoaceticus*, as well as a variety of *Acinetobacter* type strains, confirmed that CA16 clusters with the majority of *A. calcoaceticus* (Fig. 1). While the in silico DNA-DNA hybridization (*is*DDH) value between CA16 and DSM 30006 (69.3%) falls just short of the 70% value that is typically used to delineate species, CA16 is more closely related to *A. calcoaceticus* ANC 3680 (74.8%) and *A. calcoaceticus* TG19593 (73.6%).

**Fig. 2:**
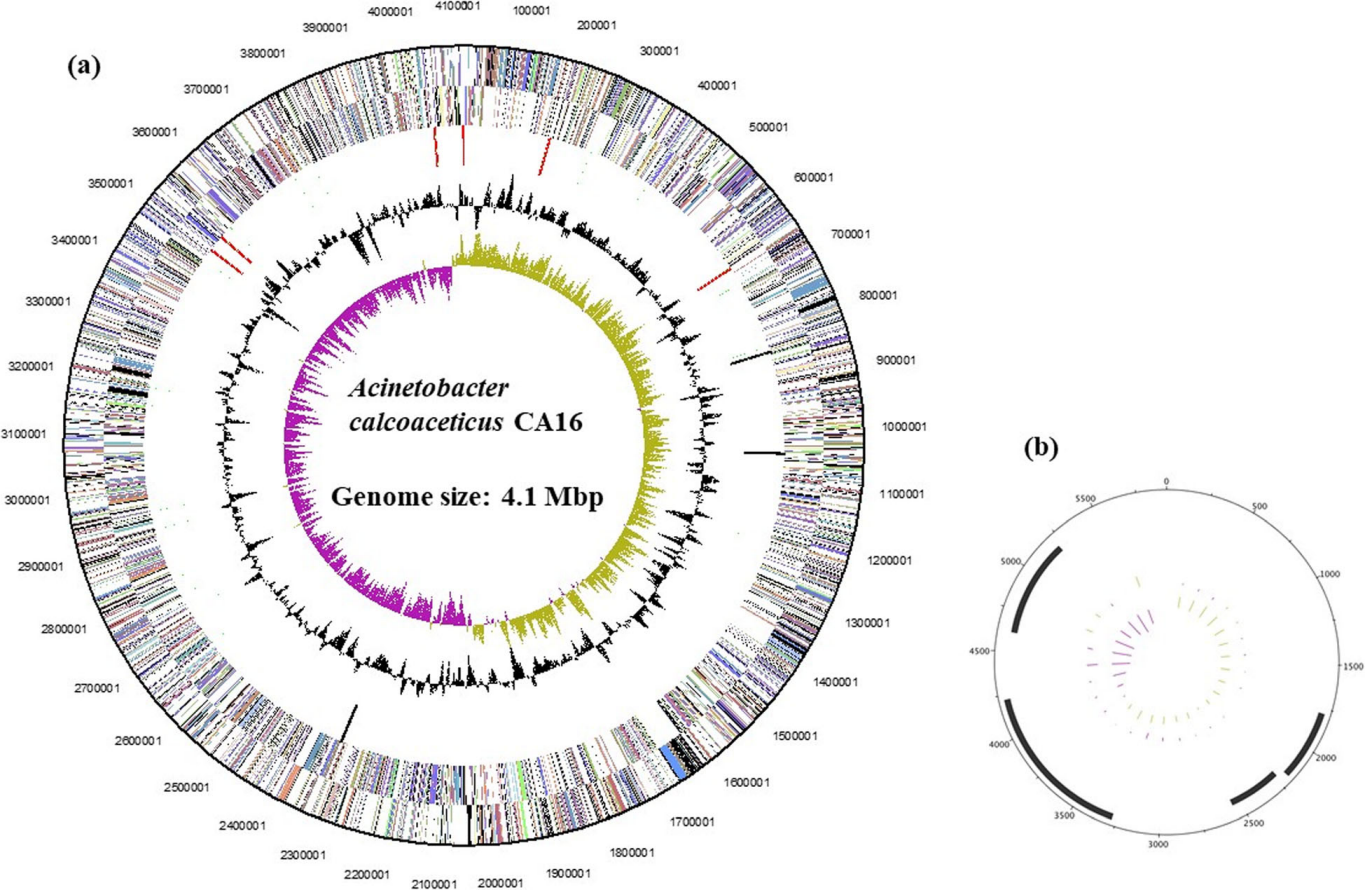
Visual representation of the completed CA16 genome. Annotated features from NCBI PGAAP and JGI IMG database were imported into Artemis and DNAPlotter. **a** *A. calcoaceticus* CA16 Chromosome. Functional categories of orthologous genes (COGs) were analyzed in the JGI IMG database. From the outside to the inside, the tracks display: CDS regions from the forward and reverse strands keyed to COG functions, RNAs (tRNAs in green, rRNAs in red, and other RNAs in black), G-C plot distribution, G-C skew and pseudogenes [29]. DNAPlotter was used to visualize **b** the CA16 plasmid. From outside to inside, the tracks display: CDS regions from the forward strand, CDS regions from the reverse strand, pseudogenes, G-C plot distribution, and G-C skew

The features of CA16 complete genomic assembly have been previously deposited to Genbank with the Accession number CP020000 and CP020001 [28]. Briefly, CA16 has a total of 3798 coding genes, 6 rRNA operons and 65 tRNAs. The annotated genome features are shown in Fig. 2. No significant metabolic genes of interest were detected on the plasmid (Fig. 2b), as most of the genes were previously established to be involved in plasmid replication [28]. The genome has 38.69% G-C content which is near the recognized range for *Acinetobactor* species (40.0 to 46.0 mol%) [30–32]. The slightly lower GC content of *A. calcoaceticus* CA16 as compared to other *Acinetobacter* may result from accumulation of AT-rich mutations due to C- > T mutation bias, or incorporation of AT-rich foreign elements such as phages, which commonly affect GC composition in bacteria [33]. Still, GC composition (whether high or low) is expected to offer some fitness advantage [34] and could possibly confer some adaptation to the specific soil microenvironment or climate in Canada.

The presented CA16 genome will facilitate downstream studies to elucidate the metabolic pathways and regulatory mechanisms implicated in bacterial hydrocarbon degradation, as well as future industrial application of the bacterial strain for sustainable environment and ecosystem.

### Growth of *A. calcoaceticus* CA16 in the presence of diesel

**Fig. 3:**
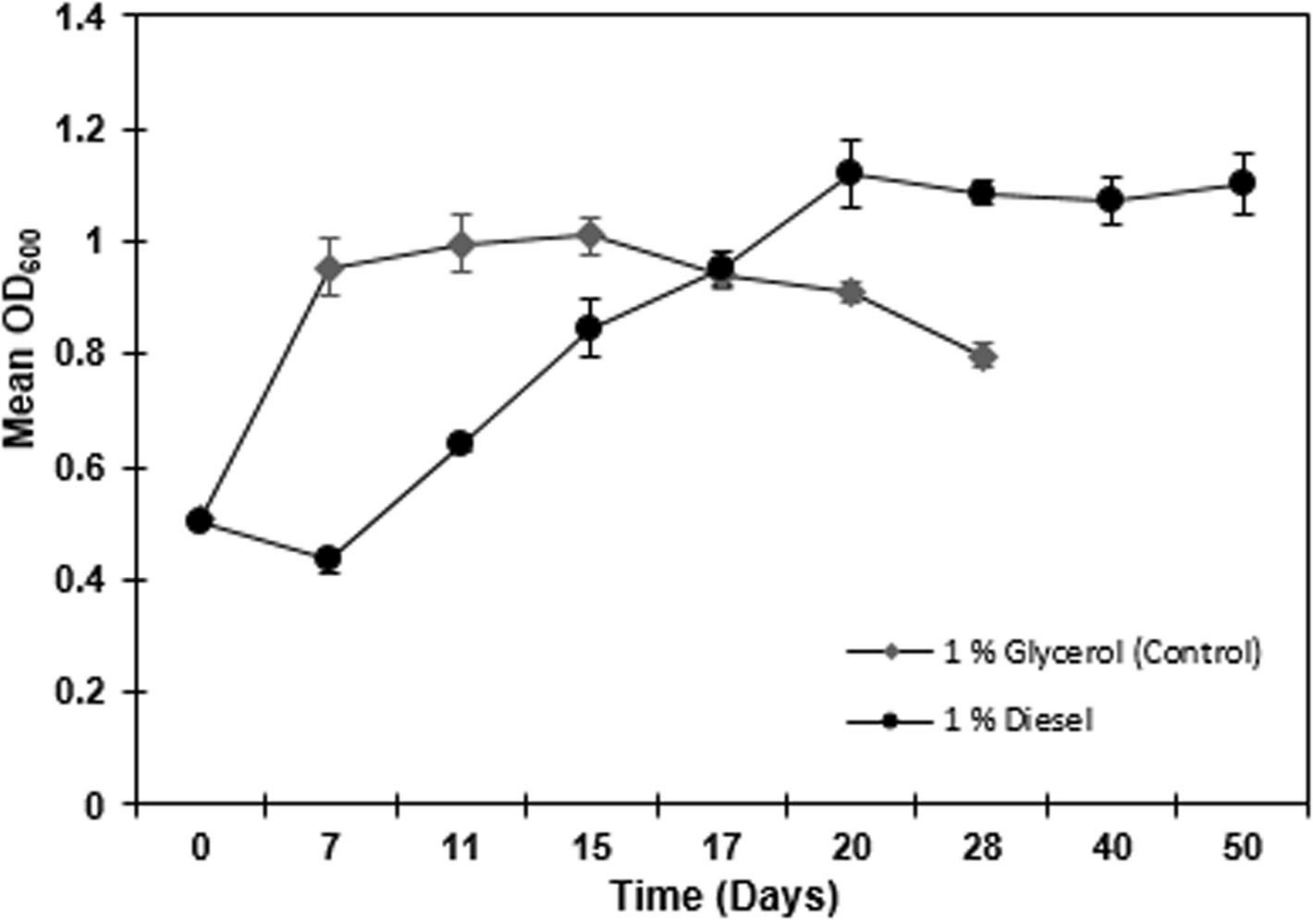
Growth curve of wild type *A. calcoaceticus* CA16 in diesel. Cells with a starting OD_600_ of 0.5 were suspended in minimal media supplemented with 1% diesel (black circles) or 0.1% glycerol (grey diamonds) as a control. The growth curve represents the mean OD_600_ ± standard error (SE) from three different biological replicates (*n* = 3)

The proliferation of wild type *A. calcoaceticus* CA16 using diesel as the sole carbon source was assessed based on optical density readings taken at 600 nm (Fig. 3). Within the first week of diesel treatment, the optical density of the bacteria decreased from the initial value of 0.5 to 0.43 ± 0.02. This indicated that some CA16 cells degraded while the culture was adjusting to the new medium (black circles, Fig. 3). However, after 7 days, the optical density of the cells had improved, reaching 1.12 ± 0.06 by Day 20. This growth phase and overall concentration of CA16 remained constant (at approximate OD_600_ of 1.1) between Days 20 to 50.

Control samples using glycerol as a carbon source saw the OD_600_ increase from 0.5 to 0.95 ± 0.05 after 1 day of treatment (grey diamonds, Fig.3). CA16 cells maintained their growth up to 3 days, followed by a slow decay after 7 days (OD_600_ = 0.9). After 15 days, the optical density of the cells approached 0.80, similar to the OD_600_ for diesel at the same treatment period. As the effect of the glycerol control improved the growth of the CA16 cells immediately after exposure, it leads us to conclude the decrease in optical densities of the CA16 in diesel samples were directly due to the diesel treatment and not a component of the media or external environment.

### Metabolomic study of diesel-degrading CA16

The metabolites produced in diesel-degradation by CA16 after 14 and 28 day treatment (growth vs. sustained growth) periods were analyzed using GC/MS. Due to the numerous peaks in the chromatograms, it was confirmed that the hydrocarbon composition of diesel (mixture of aliphatic and aromatic hydrocarbons) is extremely complex (Fig. S1).

PCA of all the diesel-treated CA16 dataset revealed the total (possible) metabolite features to be 1717. This included aliphatic and aromatic hydrocarbons and alcohols, such as 7-amino-4-methyl-2-quinolinol, 2-butyl-1-octanol and 6,10,13-trimethyltetradecanol. However, after averaging and filtering the dataset, a total of 1194 metabolite features remained (Fig. S2). This reiterates that the diesel degradation by CA16 is a highly complex process that produces a wide combination of metabolites that is likely dependent on the experimental conditions. Thus, to gain insight on the diesel degradation process, we decided to first determine if there are changes in the overall hydrocarbon content after diesel degradation by CA16 occurs.

**Fig. 4:**
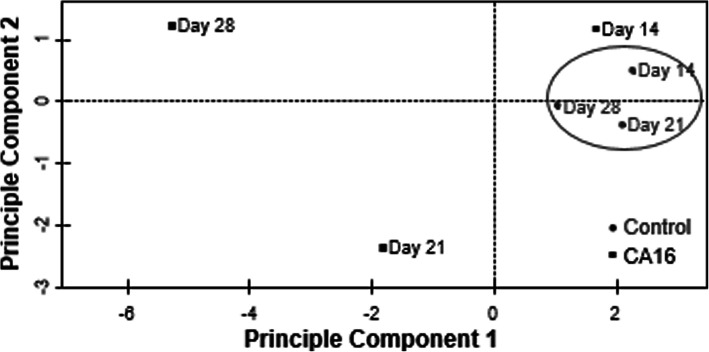
Diesel-inoculated CA16 cultures have different hydrocarbon content compared to abiotic controls. PCA of the total hydrocarbon content present in its absence of CA16 (grey circles) and after diesel degradation (black squares)

PCA of the total (remaining) hydrocarbon content confirmed significant differences between the abiotic controls and the diesel-inoculated cultures (Fig. 4). In the absence of CA16 cells, the metabolite features which remain after the diesel treatment period have very little variation present (similar products and thus, close clustering). For example, from the abiotic control (grey circles, Fig. 4), little variation can be concluded between Days 14, 21 and 28 since the 3 days were clustered closely one another with a total variance of 88%. However, diesel degradation by CA16 led to significant changes in the total end metabolite compositions since that larger variation exists between the CA16 samples and their respective treatment periods (black squares, Fig. 4). Additionally, each tested day appears to have some degree of variation as there is no obvious clustering of the PCA present. Ultimately, PCA analysis confirms are variations between the metabolites produced in the abiotic, diesel and glycerol samples. Additionally, CA16 is able to degrade (at least some of) the hydrocarbons that are present in diesel.

**Fig. 5:**
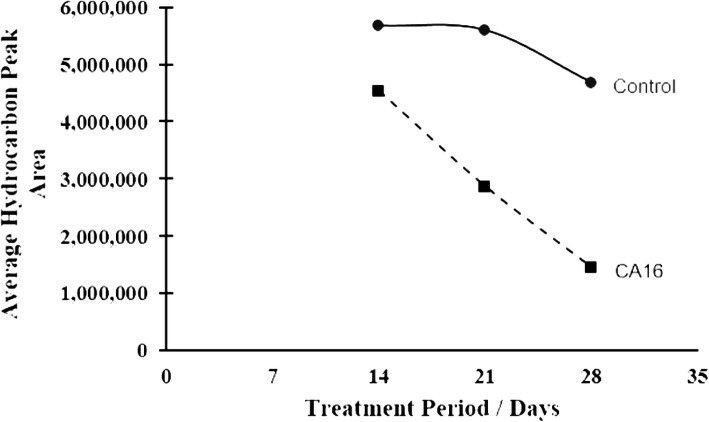
Content of total n-alkane hydrocarbons decreased after treatment with CA16. Average total peak area of aliphatic hydrocarbons after degradation by CA16 in diesel media (square markers with dashed line) and in the absence of CA16 (grey circles, solid line) at Days 14, 21 and 28

Since there were sufficient differences in the overall end products after diesel’s degradation by CA16, we decided to focus on the specific degradation effects of CA16 on the *n-*alkane (chemical formula: C_*n*_H_n + 2_, where *n* = number of carbon atoms) subclass of aliphatic hydrocarbons, which are known metabolic changes that have been associated with diesel degradation in other microorganisms [8, 13]. From analysis of the GC/MS chromatograms, we noted that a number of *n-*alkane hydrocarbons decreased in their peak area with prolonged treatment with CA16 (represented as the average total hydrocarbon peak area, Fig. 5). Here, we confirm from the control sample’s drop in the average total hydrocarbon peak area over time, that abiotic hydrocarbon loss occurs.

This is confirmation that there are reductions in diesel’s aliphatic hydrocarbon content (by CA16) and these changes are detectable by metabolomics since the total average peak area of the control samples remained relatively constant. The peaks of the aliphatic hydrocarbons were then positioned to the external C7-C40 standard and identified based on their retention times to confirm we have a mixture of C12-C24 aliphatic hydrocarbons. In this study, the focus of the CA16-degraded *n-*alkanes were: dodecane (C12), tridecane (C13), tetradecane (C14), pentadecane (C15), hexadecane (C16), heptadecane (C17) and octadecane (C18).

### Hydrocarbon degradation by CA16

**Fig. 6:**
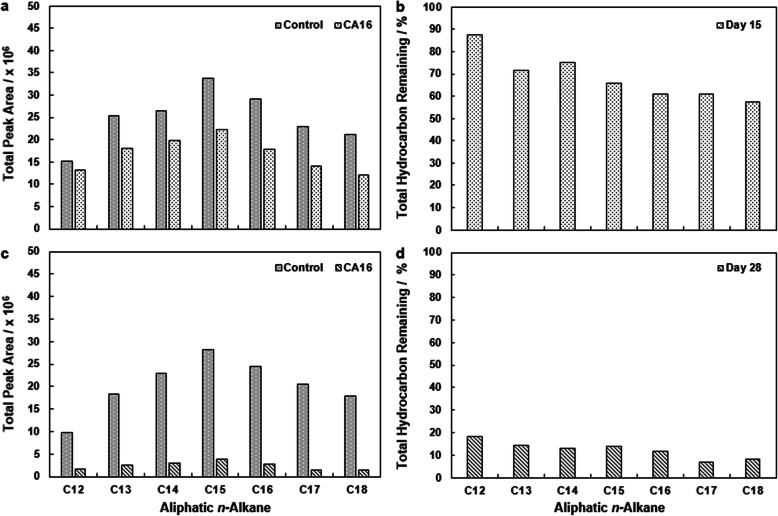
Content of specific n-alkane hydrocarbons decreased after treatment with CA16. The degradation of aliphatic hydrocarbons in 1% diesel by CA16 after (**a**, **b**) 15 and (**c**, **d**) 28 days of treatment. **a** and **c** are the plots of the total peak area from the GC/MS chromatograms and are representative of the remaining hydrocarbons after diesel-degradation, while (**b**) and (**d**) are plot representatives of the total hydrocarbon remaining after diesel degradation

From the chromatograms, comparison and analysis of the hydrocarbon peak areas indicate how much of the product remains after treatment (Fig. 6). The reduction in peak areas indicates that bacterial inoculation significantly degraded the number of hydrocarbons produced relative to the abiotic controls. After 15 days the hydrocarbon amounts were reduced by between 13% (C12) to 43% (C18) in the samples containing *A. calcoaceticus* DSCA16 as compared to the abiotic controls (average 31%). After 28 days, the hydrocarbon amounts were reduced by between 82% (C12) to 93% (C17) in the samples containing *A. calcoaceticus* DSCA16 as compared to the abiotic controls (average 88%).

### Quantification of diesel-degrading gene expression

Based on protein homology and sequence similarity, a total of eleven genes that are involved in other microorganisms’ diesel metabolism pathways was also found in CA16 (Table 1).

**Table 1:** Some diesel-associated genes present in CA16 that are similar in other microorganisms

Gene	Genome Position	Accession #	Predicted function	Most closely related RefSeq Protein	%ID
Alkane hydroxylase (*Alk*M)	1203709–1204902	AQZ81143	Alkane Monooxygenase Complex, Alk gene cluster	alkane 1-monooxygenase [Acinetobacter guillouiae] WP_004720613.1	99.45
Transcriptional Regulator (*Alk*R)	1813655–1812696	AQZ81718	Regulation of hydrocarbon degradation Alkane Monooxygenase Complex, Alk gene cluster	AraC family transcriptional regulator [Acinetobacter calcoaceticus] WP_080026973.1	100
Esterase (*Est*B)	363212–361917	AQZ80440	Esterase; Alkane Monooxygenase Complex, Alk gene cluster	MULTISPECIES: serine hydrolase [Acinetobacter] WP_075431253.1	99.77
Rubredoxin A (*Rub*A)	1004293–1004457	AQZ80981	Alkane degradation	MULTISPECIES: rubredoxin [Bacteria] WP_000760495.1	100
Rubredoxin B (*Rub*B)	1004198–1003023	AQZ80980	Alkane degradation; NAD(P)H-dependent rubredoxin reductase	MULTISPECIES: FAD-dependentoxidoreductase [Acinetobacter] WP_003653048.1	99.75
Lipoyl Synthase (LipA)	1866491–1865511	AQZ81763	Protein lipoylation	lipoyl synthase [Acinetobacter calcoaceticus] WP_080026996.1	100
Lipoyl Synthase (LipB)	3254410–3255063	AQZ82924	LipA Chaperone Protein	lipoyl (octanoyl) transferase LipB [Acinetobacter calcoaceticus] WP_005036485.1	100
Outer Membrane Protein A (*Omp*A)	861883–862536	AQZ80868	Type IV Secretion	MULTISPECIES: OmpA family protein [Acinetobacter] WP_005042643.1	100
Outer membrane lipoprotein (*wza*)	36420–35323	AQZ80175	*wee* Gene Cluster (polymer export: potential channel)	polysaccharide biosynthesis/export family protein [Acinetobacter calcoaceticus] WP_080026070.1	100
Protein tyrosine kinase (*wzc*)	34868–32688	AQZ80173	*wee* Gene Cluster (polymer export: autophosphorylation)	polysaccharide biosynthesis tyrosine autokinase [Acinetobacter calcoaceticus] WP_080026068.1	100
General secretion pathway protein E (*xcp*R)	634244–635575	AQZ80682	Secretory Protein, associated with regulating dodecane degradation	MULTISPECIES: type II secretion system ATPase GspE [Acinetobacter] WP_004641278.1	100

**Fig. 7:**
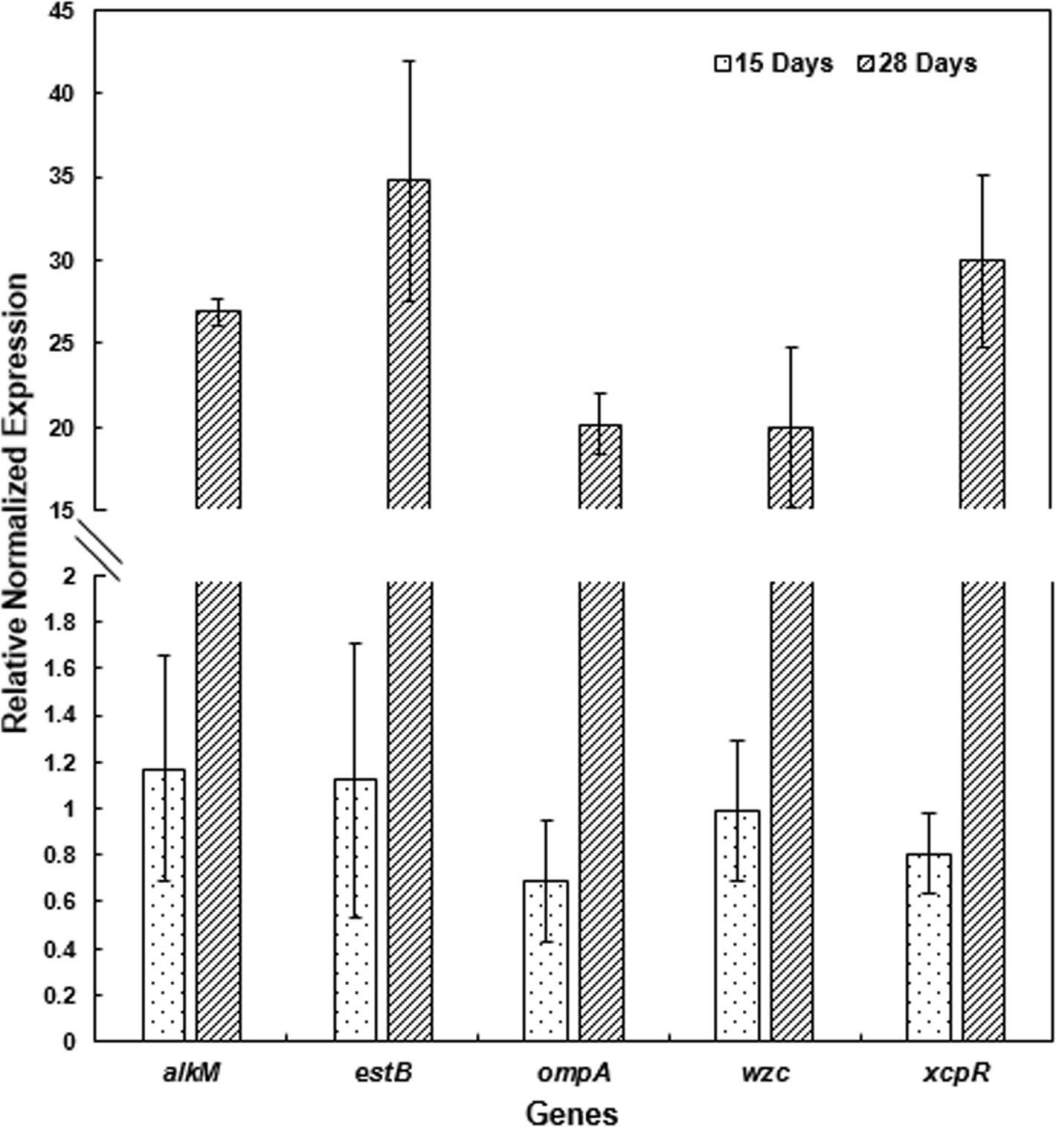
Quantitative RT-PCR analysis of the diesel inducible genes over time in *A. calcoaceticus* CA16. Cells with a starting OD_600_ of 0.5 were treated with 1% diesel or 0.1% glycerol (control). For each time point, expression of the diesel-activated genes encoding AlkM, EstB, OmpA, Wzc and XcpR (Genbank accession numbers AQZ81143, AQZ80440, AQZ80868, AQZ80173, and AQZ80682,respectively) were quantified relative to their expression in glycerol control samples. The mean expression values of four technical replicates per gene target were normalized to 16S rRNA expression. Results are presented as mean relative normalized expression ± SE of three biological replicates (*n* = 3)

To test for the presence of selected diesel-associated genes in CA16, we used quantitative RT-PCR to extract RNA from Day 15 (growth phase) and Day 28 (sustained growth phase) for 1% diesel-treated CA16 and their respective 0.1% v/v glycerol controls (Fig. 7).

On Day 15, we saw low relative normalized expressions (to the glycerol controls) of the diesel-degrading genes: *alk*M (1.17 ± 0.49), *est*B (1.12 ± 0.58), *omp*A (0.69 ± 0.25), *wzc* (0.99 ± 0.30) and *xcp*R (0.80 ± 0.17). However, on Day 28 the expression levels increased significantly: 26.89 ± 0.84, 34.73 ± 7.17, 20.17 ± 1.84, 19.96 ± 4.85, 29.92 ± 5.14, respectively. Relative to Day 15, Day 28 expression levels had increased 20 to 37 fold. These results assure CA16 diesel-degradation is actively occurring during our study.

## Discussion

### Identification of CA16

Our whole-genome phylogenetic analysis places strain CA16 in a monophyletic group with most of the other *A. calcoaceticus* strains and the type strain, *A. calcoaceticus* DSM 30006 (Fig. 1). This monophyletic group, however, includes multiple clades that could possibly represent different species based the typical DDH cutoff value of 70%. The *is*DDH values between *A. calcoaceticus* CA16 and the type strain’s clade are only just below 70% (ranging from 68.6 to 69.5, with 69.3 for the type strain DSM 30006), while for other strains in the monophyletic group the value is as low as 51.1.

*A. calcoaceticus* is known to be part of the *Acinetobacter calcoaceticus*–*Acinetobacter baumannii* complex (Acb complex), which also includes *A. baumannii*, *Acinetobacter lactucae*, *Acinetobacter nosocomialis*, *Acinetobacter oleivorans*, *Acinetobacter pittii*, and *Acinetobacter seifertii* [35]. Species within the Acb complex can not always be differentiated by phenotypic methods [36, 37], and type strains from each of these Acb complex species were included in our tree. Outside of the monophyletic *A. calcoaceticus* group, our tree predicts that currently named *A. calcoaceticus* strain ANC 3811 is actually more closely related to *A. oleivorans*, strain EGD AQ BF14 is more closely related to *A. pittii*, strains NCTC7412 and NCTC7364 are misidentified members of *Acinetobacter baumannii*, and strain JUb89 is not part of the Acb complex. The findings with strains EGD AQ BF14 and NCTC7364 are consistent with a previous phylogenetic analysis of *Acinetobacter* that included fewer strains of *A. calcoaceticus* [35]. Of interest, in this previous analysis, strain ANC 3811 (which groups with *A. oleivorans* in our tree) formed a basal clade within *A. calcoaceticus* in a tree based on 225 single gene families but was grouped with *A. oleivorans* in a tree based on 13 top marker genes [35]. Our tree otherwise agreed with the evolutionary relationships presented in the 225 single gene family tree for the included *A. calcoaceticus* strains, but our study agreed instead with the 13 top marker gene tree in terms of the relationships between *A. baumannii*, *A. nosocomialis*, and *A. seifertii*.

### Growth in diesel

*A. calcoaceticus* CA16 was able to grow in minimal media supplemented with 1% diesel. These results suggest that CA16 can use diesel as a carbon source to promote growth. Since we did not use a control without a carbon source, we can not rule out the possibility that CA16 can fix carbon from the atmosphere, although our preliminary analyses indicate that this is not the case.

Looking at growth of *A. calcoaceticus* CA16 over time, using diesel as the sole carbon source (Fig. 3), the isolate initially suffered a lag phase, likely due to the removal of a favorable carbon source (terrific broth to diesel-supplemented minimal media). By day 11, growth had resumed with and at Day 15, both the diesel and glycerol control treatments had similar OD_6oo_ values (approximately 0.8), indicating the amount of cells present were similar between samples.

After Day 15, the OD_600_ of the glycerol control began to decrease while the diesel-treated CA16 cells were still thriving. This is likely due to depletion of the necessary nutrients in the glycerol medium. Nutrient depletion would occur earlier in the glycerol treatment than diesel treatment due to glycerol (C_3_H_8_O_3_) being readily metabolized in cells and supporting quicker progression through the growth curve. There is also the possibility that the buildup of toxic metabolite byproducts occur earlier or more quickly in CA16 culture after glycerol exposure. Upon longer diesel treatment (Days 20 through 50), the growth rate of the CA16 isolates appeared to have stabilized and plateaued to maintain its overall cell densities. This is similar to a previous study by Sepic et al. [38] on *Pseudomonas fluorescens,* Texaco, which had greater biodegradation of aliphatic hydrocarbons in diesel oil within the first 20 days of experimentation.

### Metabolomics

In order to further understand this bacterial strain’s ability to degrade diesel hydrocarbons, a metabolomic analysis of the final extracts was conducted. Since the total metabolite features numbered into the thousands, we first confirmed there was overall (and detectable) differences in the final hydrocarbon content of the diesel media after treatment with CA16 (Fig. 4), followed by narrowing our focus to the aliphatic C12-C18 hydrocarbons (Fig. 5). Additionally, this allows us to focus on a hydrocarbon subclass that has previously reported significant degradation (by *Pseudomonas fluorescens,* Texaco) within the first 20 days [38].

Our results showed that *A. calcoaceticus* CA16 degraded the diesel hydrocarbons on Day 15 by an average of 31% (range 13 to 43% for C12 and C18, repectively). On Day 28, it degraded the hydrocarbons by an average of 88% (range 82 to 93% for C12 and C17, respectively). The percent degradation of each hydrocarbon had a lower range than for some other *Acinetobacter*, such as *Acinetobacter junii* VA2 which removed 91.7% of C18 but only 0.2% of C16 after 15 days [39].

The performance of various *Acinetobacter* for diesel degradation, when diesel is used as the sole carbon source, is shown in Table 2. Comparatively, CA16 shows a good ability to degrade diesel although the time taken to do so is longer than for many of the other listed strains. Accurate comparisons are difficult, however, since the presented studies used different starting amounts of diesel and cells, as well as different media components and temperatures. Some of these studies also show that drastic improvements can be obtained with a single strain, for example by adding nutrients [14] or surfactants [41], or by immobilizing cells [43].

**Table 2:** Diesel biodegradation by single *Acinetobacter* isolates without additional carbon source

Species	% Diesel degraded	Timeline	Initial diesel	Initial culture	Ref.	Notes
*A. calcoaceticus* CA16	88%	28 days	2%	OD_600_ = 0.5	present study	
*A. calcoaceticus* GK2	87%	10 days	0.1% (1 g/L)	OD_590_ = 0.1	[40]	
*Acinetobacter oleivorans* PF1	16%	10 days	0.1% (1 g/L)	OD_590_ = 0.1	[40]	
*Acinetobacter haemolyticus* 2SA	70%	21 days	1%	unknown	[41]	90% degradation with the biosurfactant kurstakin
*Acinetobacter baumannii*	58.1%	10 days	2%	unknown	[42]	Culture OD_600_ was 1.515 after 10 days
*Acinetobacter venetianus*	76%	4 days	0.02% (200 mg/L)	OD_600_ = 0.7	[43]	90% degradation with immobilized cells
*Acinetobacter junii* VA2	75.8%	15 days	0.05% (0.5 mg/mL)	5 × 10^8 cells/mL	[39]	
*Acinetobacter beijerinckii* ZRS	20.87%	7 days	0.5%	OD_600_ = 0.05	[14]	80.40% degradation with nutritional supplements including yeast extract
*Acinetobacter* sp. strain Y2	80%	10 days	2%	4 × 10^7 cells/mL	[44]	
*Acinetobacter haemolyticus* MJ01	93.3%	3 days	0.1% (1000 mg/L)	6 × 10^6 CFU/mL	[45]	
*Acinetobacter johnsonii* MJ4	92.9%	3 days	0.1% (1000 mg/L)	6 × 10^6 CFU/mL	[45]	

Additionally, our metabolomic study predicts depletion of the aliphatic hydrocarbons is likely to occur with longer treatment (Fig. 5). Literature suggests once the aliphatic hydrocarbons from diesel are depleted, the remaining carbon sources for the bacteria would be the higher molecular mass branched hydrocarbon compounds that are less susceptible to abiotic loss and biodegradation (earlier on) [38].

Across the tested experimental conditions, the overall distribution of total hydrocarbons remains mostly the same: the C15 levels that remained in the culture broth was the highest among the hydrocarbons, while the minimum and maximum chain lengths, C12 and C18, had the least. The only exception occurred on Day 28, where C17 had the least amount remaining. This indicated that CA16 degraded more C17 than C18 alkane after 4 weeks had passed. The consistency among the distribution pattern suggests that there was no change in degradation preference for C12-C16 *n-*alkanes as the treatment period lengthened. It also appears that although CA16 is in different growth phases on Days 15 and 28 (growing vs sustained growth), the hydrocarbon reduction abilities of CA16 appear to improve (amount reduced is over doubled) since the optical densities (concentration) of the CA16 did not change drastically (0.85 (Day 15) vs. 1.09 (Day 28)). Unlike some other individual bacterial strains, our isolated *A. calcoaceticus* CA16 strain appears to be able to reduce a wide range of aliphatic *n-*alkanes (C12-C18).

Therefore, the length of the carbon chain appears to have some influence over the CA16’s degradation ability. It is likely that the longer hydrocarbon *n-*alkanes are metabolized into the smaller hydrocarbon chains since the first step in biodegradation of *n-*alkanes is oxidization of the hydrocarbon into its corresponding alcohol [6]. In most cases, anaerobic bacterial degradation of *n-*alkanes occurs by oxidizing a terminal methyl group off the hydrocarbon chain to generate a primary alcohol [46, 47]. This alcohol gets further oxidized to the corresponding aldehyde and finally, converted to a fatty acid, which becomes incorporated into the β-oxidation cycle [32, 48]. If the longer chain hydrocarbons are indeed being oxidized into an alcohol and a shorter chain hydrocarbon, it is likely these short chain hydrocarbon products are contributing to the total hydrocarbon content that remains after treatment. However, it is also recognized that a dioxygenase can convert the *n-*alkane directly into its aldehyde form through *n-*alkyl hydroperoxidase (the Finnerty pathway) [32, 49, 50]. Additionally, researchers have found that *n-*alkane oxidation in some *Acinetobacter* species can occur without the alcohol intermediate [49, 50]. The metabolomic study of CA16 degrading diesel does indicate the presence of some alcohols, however a further in-depth study is required to determine the mechanism of diesel degradation by CA16.

### Activation of genes

While several scientific reports (and our own results) suggest that *Acinetobacter* species can degrade diesel oil efficiently [14, 15], there are only few studies to explain the regulation in gene expression during diesel degradation by bacteria [51–53]. To understand the gene expression levels produced in *A. calcoaceticus* CA16 during the diesel degradation process, we studied selected genes that are involved in hydrocarbon degradation (Table 1). Using qRT-PCR, we found several diesel-degrading genes increased in CA16 after treatment with 1% diesel: *alkM, estB, ompA, wzc* and *xcpR*.

On Day 15, we detected a relative normalized expression of 1.17 ± 0.49 for *Alk*M, which increased to 26.89 ± 0.84 on Day 28 and 1.12 ± 0.58 to 34.73 ± 7.17 for EstB. Specifically, these genes are responsible for alkane hydroxylase (alkM), alkane monooxygenase complex and esterase (estB). With 23- and 31- fold elevated *AlkM* and *EstB* expressions, respectively, we can confidently confirm higher hydrocarbon degradation occurs later in the treatment. These alkane hydroxylases or monooxygenases will oxidize the *n-*alkane, whether terminal, sub terminal or bi terminal (Ji et al., 2013), to convert it to an alcohol. Longer chained alkanes often require P450 cytochrome complexes or different hydroxylase enzymes to process (van Beilen and Funhoff, 2007; Wang and Shao, 2013). Subsequent carboxylation into a fatty acid by alcohol or aldehyde dehydrogenases prepares the molecule for oxidation.

After 28 day diesel treatment, three additional genes were activated and expressed at normalized levels significantly higher than the control glycerol-degradation by CA16: *ompA* (20.17 ± 1.84)*, wzc* (19.96 ± 4.85) and *xcp*R (29.92 ± 5.14). These genes are responsible for wee gene cluster (polymer export, *ompA*), regulating dodecane degradation secretory pathway (*wzc*) and dodecane degradation and outer membrane protein A (secretion, *xcp*R) [54]. Both *wzc* and *xcp*R also have roles in dodecane degradation and thus, activation and higher expression of the genes are expected if dodecane degradation in CA16 is occurring. Interestingly, on Day 15 these genes expressed lower to similar levels as the glycerol controls (*ompA* 0.69 ± 0.25; *wzc* 0.99 ± 0.30 and *xcp*R 0.80 ± 0.17). From our genomics study, we were able to confirm the activation and expression of these genes: a 20- and 37-fold increases in *wzc* and *xcp*R gene expressions were detected (Day 28 vs 15). Our metabolomic study indicated the overall reduction in C12 content from the diesel-supplemented media was 82% (relative to the abiotic control) after 28 days and 13% after 15 days (Fig. 6). This correlated to almost 6.5-fold improvement in the total reduction of dodecane levels after prolonged treatment with CA16. Overall, qRT-PCR confirmed the expression of our selected diesel-degrading genes: *alkM*, *est*B, *omp*A, *wzc* and *xcp*R in CA16 for both of the tested treatment lengths. After 28 days, the genes expressed between 20- to 37-fold higher than their Day 15 counterparts. Collectively, our genomics and metabolomic study highly complement each other to demonstrate CA16’s diesel-degrading ability. These results complement another recently published study by Hassan et al., who studied the solar-bioelectrokinetics of soil contained with heavy petroleum hydrocarbons by *A. calcoaceticus*-16 [55]. Hassan et al. found genes encoding 1698/2041, RubA, LipA, LipB, Alk-b2 and P450 present in the microbe. Lipase enzymes, LipA and LipB, and alkane 1-monogenase 2 (alkB2) are known to be involved in the degradation of diesel (Table 1). In our future experiments, we plan to use transcriptomic analysis to gain a more comprehensive view of CA16 gene expression during degradation of diesel.

The application of hydrocarbon-degrading bacteria in diesel-contaminated sites may not guarantee that all unwanted hydrocarbon components will be completely metabolized, since some components, such as short or long chain alkanes (<C_10_ and C_20_-C_40_) are not as readily biodegradable [32, 56, 57]. For example, *Acinetobacter* strains isolated by Kubota et al. were able to degrade *n-*hexadecane (C16) and *n-*icosane (C20) but could not degrade other *n-* or cyclic alkanes [58]. Since our isolated strain CA16 can degrade a wide range of *n-*aliphatic alkanes (C12-C18) that are present in diesel, utilizing the strain in microbial consortiums for bioremediation could prove to be highly effective. As microbial consortia are designed based on the strain properties and the ability to work synergistically with other microbes, it becomes necessary to first study CA16 individually to provide mechanistic insight into the diesel-bioremediation process.

## Conclusions

Here, it was demonstrated that *A. calcoaceticus* CA16 is capable of utilizing diesel as the sole source of carbon and energy through degradation of its aliphatic hydrocarbons. The genome analysis of *A. calcoaceticus* CA16 (NCBI Genbank accession CP20000.1 and CP20001.1) verifies its genetic basis for these properties. Unlike some of the other members of the *Acinetobacter* genus, our isolated CA16 appears to efficiently degrade aliphatic alkane hydrocarbons (82 to 92% in 28 days; C12 through C18). These results suggest that strain CA16 has good potential for bioremediation of diesel oil polluted environments. Since *A. calcoaceticus* CA16 was isolated from a soil environment in Southern Ontario, Canada, it may be particularly useful for terrestrial spills in this location.

## Methods

### Chemicals

Diesel (comprised mostly of C12-C20 alkanes) was obtained onsite at Agriculture and Agri-Food Canada (London, ON) and was syringe filtered for sterility using a 0.2 μm Supor Membrane (PALL Corporation, Mississauga, ON). Bacterial cultures were grown in Terrific broth (tryptone 12 g/L, K_2_HPO_4_ 9.4 g/L KH_2_PO_4_ 2.2 g/L, yeast extract 24 g/L and 4 mL/L glycerol) and substituted with minimal media (NaCl 6 g/L,(NH_4_)_2_SO_4_ 1 g/L, KH_2_PO_4_ 0.5 g/L, K_2_HPO_4_ 0.5 g/L, MgSO_4_·7H_2_O 0.1 g/L, CaCl_2_ 0.1 g/L and MES hydrate 1 g/L), that was supplemented with either diesel or glycerol as the sole carbon source. *N,*O-Bis (trimethylsilyl) trifluoroacetamide with 1% Trimethylchlorosilane (BFTSA-TMCS) was purchased from Supelco (Bellfonte, PA). Glycerol (≥99.5%), dichloromethane (DCM, HPLC grade), anhydrous sodium sulfate, squalene and C7-C40 saturated alkanes standards were purchased from Sigma Aldrich (Mississauga, ON). All chemicals were used without further purification.

### Isolation of *A. calcoaceticus* CA16

Previously, canola roots from southwestern Ontario were harvested by our group [28] to isolate their microbes. The sample collection followed standard guidelines required by Environment Canada, and research with the bacterial strains was done in accordance with biosafety procedures at Agriculture and Agri-Food Canada. First, the roots were rinsed well with sterilized deionized water to remove soil contaminants, followed by fine grinding by a mortar and pestle. The root grinds were then suspended in 0.85% saline solution. Nitrogen-fixing agar plates were inoculated with serial dilutions of the canola root solution to identify potential nitrogen-fixing microbes of interest. These isolates were then screened on minimal media supplemented with 0.2% diesel for their diesel-degrading abilities. The isolate which had the largest colony and fastest growth rate on diesel media was selected for genomic identification (later identified as ‘CA16’). Isolated CA16 colonies were cultured in Terrific broth and agitated overnight at 28 °C. Cells were then pelleted in a high-speed centrifuge at 9400 x g for 10 min and the supernatant discarded. The cell pellets were washed twice with sterile 0.85% saline solution before re-suspension in minimal media for experimentation.

### Genomic analysis

Genomic DNA was extracted from the bacterial isolate using the GenEluteTM Bacterial Genomic DNA Kit (Sigma-Aldrich, Mississauga, ON). Extraction followed the manufacturer’s protocol with the following modification: UltraPure™ DNase/RNase-Free Distilled Water (Life Technologies, Grand Island, NY, USA) was used in place of the GenEluteTM elution buffer. Genomic DNA was sequenced on the Illumina NextSeq500 platform [28].

The draft genome was aligned to test against reference genomes on Mauve (version 2.4.0), the multiple genome alignment software [59]. Sequences of closely related species and strains were downloaded from the National Center for Biotechnology Information (NCBI) Genbank (accession numbers CP002177.1, CP009257.1, and CP010368.1 for *Acinetobacter pittii*, *Acinetobacter baumannii*, and *Acinetobacter nosocomialis*, respectively) to generate a preliminary chromosome map that served as a guide to orient the various contigs. Gaps missing from the initial suggested alignments from Mauve were amplified with PCR and sequenced by the Sanger method. Alignment of amplified sequences was performed with Lasergene Seqman Pro by DNASTAR. The completed genome was previously submitted to NCBI GenBank (Accession CP020000 and CP020001) [28] and annotated through the Prokaryotic Genome Annotation Pipeline. Functional categories of orthologous genes (COGs) were analyzed in the JGI IMG database. Annotated features from NCBI PGAAP and JGI IMG database were imported into Artemis and DNAPlotter [60].

### Identification and phylogenetics

The CA16 genome sequence data was uploaded to the Type (Strain) Genome Server (TYGS), available at https://tygs.dsmz.de, for a whole genome-based taxonomic analysis [61]. Briefly, closely related type strains were identified by extracting the 16S rDNA gene sequence and BLASTing against the 16S rDNA gene sequence of each of the currently 9856 type strains available in the TYGS database. The 50 best matches (according to the bitscore) were used to calculate precise genome distances using the Genome BLAST Distance Phylogeny (GBDP) approach under the algorithm ‘coverage’ and distance formula d5 [62].

After identifying the closest type strain as *A. calcoaceticus*, the process was repeated by including assembly accession numbers for all 19 *A. calcoaceticus* genomes currently available at NCBI (excluding the type strain which is already available in TYGS) and one *A. lactucae* strain that showed relatively high genome similarity to CA16 (39.4%) compared to other non-*A. calcoaceticus* strains.

For phylogenetic analysis through TYGS, pairwise comparisons among the input assemblies and closely related type strains (identified as above) were inferred using distance formula d5. One hundred distance replicates were calculated for each. Digital DDH values and confidence intervals were calculated using the recommended settings of the GGDC 2.1 [62]. The resulting intergenomic distances were used to infer a balanced minimum evolution tree with branch support via FASTME 2.1.4 including SPR postprocessing [63]. Branch support was inferred from 100 pseudo-bootstrap replicates each. The tree was visualized using PhyD3 [64] and adapted in PowerPoint (Microsoft Corporation, Redmont, WA, USA). The tree was rooted with *Acinetobacter brisouii*, which is known to be outside of the Acb complex, and forms part of a basal cade in the *Acinetobacter* phylogeny [35].

Full-genome in silico DNA-DNA hybridization (*is*DDH) was determined by the GGDC web server available at http://ggdc.dsmz.de/ using Formula 2 (or formula *d*_4_), which is the recommended formula particularly when dealing with extrachromosomal DNA (e.g. plasmids) and genome rearrangements [62].

### Plant growth-promoting properties

The nitrogen fixation ability of the isolate was tested by growing the bacterial cultures in nitrogen free media (KH_2_PO_4_ 0.4 g/L, K_2_HPO_4_ 0.1 g/L, MgSO_4_·7H_2_O 0.2 g/L, NaCl 0.1 g/L, CaCl_2_ 0.02 g/L, FeCl_3_ 0.01 g/L), Na_2_MoO_4_·2H_2_O 0.002 g/L), while CA16 cultures were prepared on agar plate assays (NaCl 6 g/L, NH_4_SO_4_ 1 g/L, KH_2_PO_4_ 0.5 g/L, K_2_HPO_4_ 0.5 g/L, MgSO_4_·7H_2_O 0.1 g/L, CaCl_2_ 0.1 g/L, MES hydrate 1 g/L, 15 g/L agar) to test their ability to fix carbon dioxide. To test CA16’s ability to solubilize inorganic phosphate and potassium, National Botanical Research Institute’s phosphate growth and Alexandrov’s media were used, respectively [65, 66]. Bacterial ability to produce nanocellulose was tested using Congo red [67]. After 7 days of incubation at 28 °C, the presence of a zone of clearance or growth indicated positive results for the plant growth-promoting characteristics.

### Growth of CA16 in diesel-supplemented media

For diesel degradation experiments, CA16 was cultured in minimal media supplemented with diesel as the sole carbon source. The initial OD_600_ was adjusted to 0.5 for growth curves and degradation experiments. 30 mL of CA16 cells suspended in minimal media were distributed into sterile screw-capped Kimex glass tubes (126 mm × 29 mm, Kimble Chase, Rockwood, TN, USA) to characterize their growth in 0.25, 0.5, 1 or 2% diesel or 0.1% v/v glycerol (positive control). All experiments were repeated in triplicates (*n* = 3). Diesel- and glycerol-supplemented cultures were incubated for 14, 21 and 28 days on a drum rotor (30 rpm) at 28 °C. Abiotic controls containing 20 ml of minimal media and 1% diesel were incubated alongside to account for abiotic hydrocarbon loss during experimentation.

### Metabolome analysis

Metabolites were extracted using a 1:1 liquid-liquid extraction [68]. Briefly, 30 mL of the cultures from the diesel degradation experiments were harvested after 14, 21 and 28 days. Metabolites from the aqueous cultures were collected using DCM and treated with anhydrous sodium sulfate to remove any remaining aqueous solution. The metabolome samples were dried using a rotovap, filter-sterilized and re-suspended in 1 mL of DCM for metabolomic analysis.

### Gas chromatography/mass spectrometry (GC/MS) analysis

Samples were run on a gas chromatograph 7890A coupled to a 5975C mass spectrometer (Agilent Technologies Inc., Santa Clara, CA, USA) that is equipped with a 5% phenyl methyl siloxane column (10 m duraguard + 30 m × 250 μm i.d. × 0.25 μm-film thickness, Agilent Technologies Inc.). Helium was used as the carrier gas at a constant rate of 1 mL/min. The 82 min method injected 1 μL of sample at a 1:25 split ratio. The initial oven temperature of 70 °C was sustained for 2 min, followed by an increase to 280 °C at a rate of 3 °C/min. This temperature was sustained for 1 min prior to the oven temperature being increased to 310 °C at a rate of 20 °C/min. The final temperature was held for 7.5 min to clean the column. Samples were derivatized with 100 μL BFTSA-TCMS in a 60 °C water bath (1 h). For quantification of diesel reduction, 1 μL of 0.5 ppm of squalene was added to each sample as an internal standard.

Raw Agilent Technologies data files were converted into CDF files using Chemstation and processed through xcms [69] and Metabox [70]. The chromatographic peaks were aligned based on the internal standard C7-C40 and deconvoluted using XCMS in R. Principle component analysis (PCA) was performed to visualize sample variance and differences. To identify metabolites, mass to charge ratio (m/z) and retention time of aligned peak lists were referenced to standard spectra from the 2011 National Institute of Standards Technology (NIST) library.

### Gene expression analysis by quantitative reverse-transcription PCR

Cell pellets were collected and snap-frozen in liquid nitrogen. Total cell RNA was extracted using the Geneaid Presto™ Bacterial RNA Extraction Kit (Geneaid Biotech Ltd., New Taipei City, Taiwan) and yields were measured on a Thermo Fisher Scientific NanoDrop 2000 Spectrophotometer (ThermoFisher Scientific, Burlington, ON). cDNA was generated using 150 ng of RNA per sample with SuperScript™ III Reverse Transcriptase (ThermoFisher Scientific) according to the manufacturer’s instructions.

qPCR was performed using the Bio-Rad SoFast™ EvaGreen® Supermix on the Bio-Rad CFX96 Touch Real-Time PCR Detection System (Bio-Rad Laboratories (Canada) Ltd., Mississauga, ON) with primers for the genes *alkM* (5′-CCATTTCCGAATTGAACACC-3′ and 5′-TGCCCATTTTCTTTCTTTGC-3′), *estB* (5′- TGCGAGATGAGAACAACCTG-3′ and 5′-GAACATCGCTGGAGCAAAAT-3′), *ompA* (5′- GTGACCTTCGATACGTGCAG-3′ and (5′-AATGCTGGTTTTGGTGCTTT-3′), *wzc* (5′-AGCCGAATCTGCACAAACTT-3′ and 5′-CCTCAACCTCGCGATACAAT-3′), and *xcpR* (5′-GCGATAGCAGGGACTAATGC-3′ and 5′-TCTGCTGAAGGCACATCAAG-3′). Genbank accession numbers for AlkM, EstB, OmpA, Wzc, and XcpR are AQZ81143, AQZ80440, AQZ80868, AQZ80173, and AQZ80682,respectively. Reactions were amplified in technical replicates of four (*n* = 4) and detected with SYBR® Green I. Each gene target used the following cycle: 95 °C for 30 s to denature, forty cycles alternating at 95 °C for 5, followed by 65 °C for 10 s, 95 °C for 10 s, and 65 °C for 5 s. The raw output was analyzed on Bio-Rad CFX Manager 3.1 using 16S RT-PCR primers (5′-ACCAGCTCTTGACATTCGGG-3′ and 5′-GTCCCCTTAGAGTGCCCAAC-3′) to normalize the gene expression data. Normalized expression was calculated by the Bio-Rad CFX Manager program.

## Supplementary information

**Additional file 1.**

**Additional file 2.**

## Data Availability

The datasets used and/or analysed during the current study are available from the corresponding author on reasonable request.

## Abbreviations

Acb complex: *Acinetobacter calcoaceticus*–*Acinetobacter baumannii* complex
CA16: *Acinetobacter calcoaceticus* CA16
GC/MS: Gas chromatography/mass spectrometry
NIST: National Institute of Standards Technology
OD_600_: Optical density at 600 nm
qRT-PCR: Quantitative Reverse-Transcription PCR
PCA: Principle component analysis
SE: Standard error
