# Supplementary figures and images for: Characterization and genomic analysis of a diesel-degrading bacterium, Acinetobacter calcoaceticus CA16, isolated from Canadian soil
Source: BMC Biotechnol. 2020 Jul 25;20:39. doi: 10.1186/s12896-020-00632-z (PMC7477861; doi:10.1186/s12896-020-00632-z)

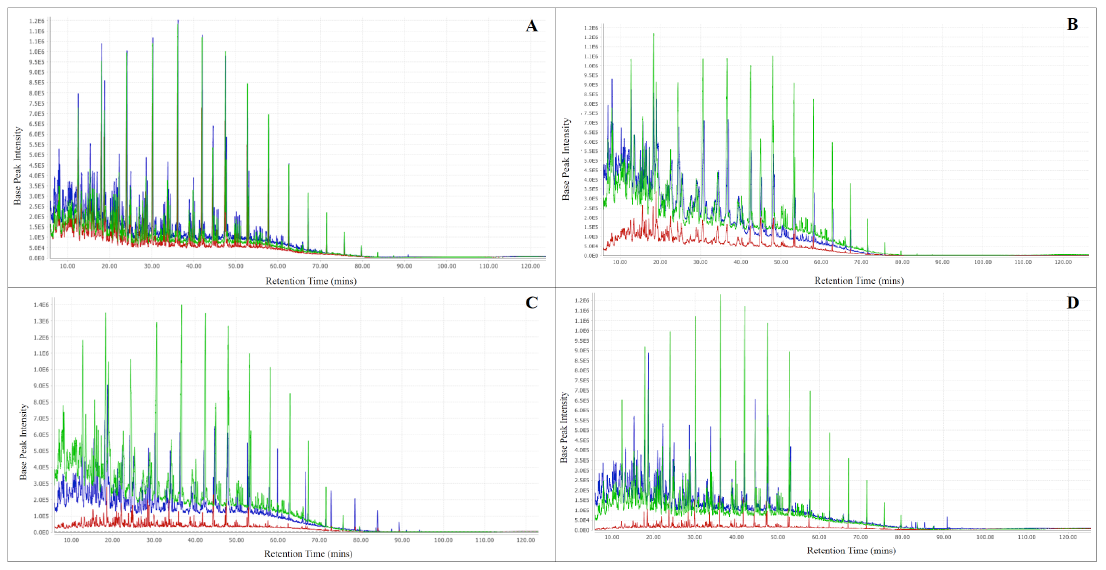

Supplement: Supplementary file 1 — Additional file 1. [file 12896_2020_632_MOESM1_ESM.png]

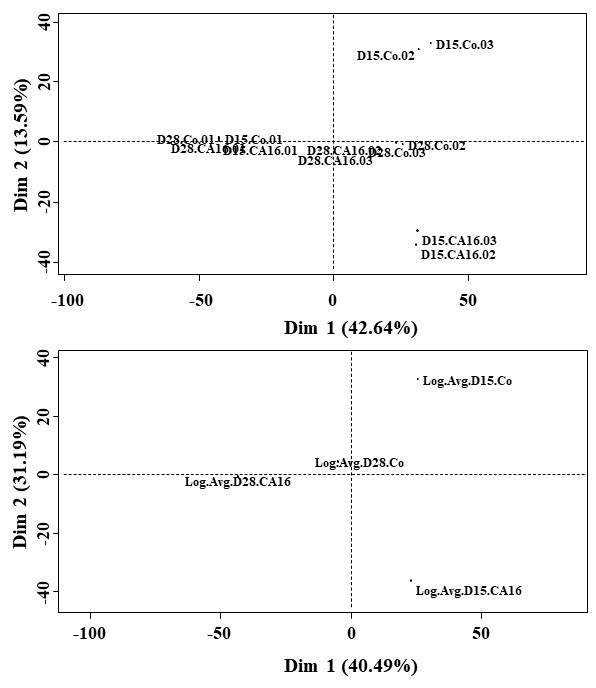

Supplement: Supplementary file 2 — Additional file 2. [file 12896_2020_632_MOESM2_ESM.jpg]
